# High expression of a disintegrin and metalloproteinase-9 predicts a shortened survival time in completely resected stage I non-small cell lung cancer

**DOI:** 10.3892/ol.2013.1209

**Published:** 2013-02-22

**Authors:** JUN ZHANG, JUAN QI, NING CHEN, WEINENG FU, BAOSEN ZHOU, ANGUANG HE

**Affiliations:** 1 Department of Molecular Targeted Therapeutics, China Medical University Lung Cancer Center, The First Hospital of China Medical University, Shenyang, P.R. China; 2 Department of Logistic Management, College of Economics and Management, Liaoning University of Traditional Chinese Medicine, Shenyang, P.R. China; 3 Department of Thoracic Surgery 1, China Medical University Lung Cancer Center, The First Hospital of China Medical University, Shenyang, P.R. China; 4 Departments of Molecular Genetics, China Medical University, Shenyang, P.R. China; 5 Epidemics, China Medical University, Shenyang, P.R. China; 6 Pathology, China Medical University, Shenyang, P.R. China

**Keywords:** ADAM9, lung neoplasm, immunohistochemistry, prognosis, lobectomy

## Abstract

The aim of this study was to investigate the abnormal expression of a disintegrin and metalloproteinase-9 (ADAM9) in human resected non-small cell lung cancer (NSCLC) tissue, in order to evaluate the significance of ADAM9 expression in surgically resected NSCLC. Sixty-four cases of completely resected stage I NSCLC with mediastinal N2 lymph node dissection were immunohistochemically analyzed for ADAM9 protein expression. Survival, univariate and multivariate analyses were conducted to assess the significance of ADAM9 expression and its correlation with other clinicopathological characteristics. ADAM9 was observed to be significantly more highly expressed in NSCLC tissue compared with normal control lung tissue (P=0.001). The 5-year survival rate for patients with NSCLC tissues highly expressing ADAM9 was significantly lower when compared with NSCLC tissues of patients exhibiting low expression of ADAM9 (56.9 vs. 88.9%, P= 0.012). Multivariate analysis identified that high expression of ADAM9 is an independent factor of shortened survival time in resected stage I NSCLC (HR, 3.385; 95% CI, 1.224–9.360; P=0.019). These results clearly demonstrate that ADAM9 is highly expressed in NSCLC and highly expressed ADAM9 correlates with shortened survival time, suggesting that ADAM9 is a novel biomarker for predicting prognosis in resected stage I NSCLC. ADAM9 may also become a useful predictive biomarker for the selection of adjuvant chemotherapy treatment of NSCLC.

## Introduction

Lung cancer is the leading cause of cancer-related mortality in China and the United States. Globally, the overall 5-year survival rate is still as low as 10–16% (1,2). Non-small cell lung cancer (NSCLC) accounts for 85% of all lung cancer cases, and surgical resection remains the most successful option for cure for patients with operable NSCLC (1,2). The 5-year survival rate for resected stage I NSCLC ranges between 60–70%, however 30–40% of early-stage patients die within 5 years of surgery, mostly as a result of metastatic disease presenting at the time of surgical resection (3,4). Investigating the genes involved in the processes of cell invasion and metastasis in NSCLC, as well as understanding and elucidating their molecular biological mechanisms, are essential steps for the diagnosis, treatment and prognosis prediction of NSCLC.

A disintegrin and metalloproteinases (ADAMs) are type I transmembrane proteins containing both a metalloproteinase and disintegrin extracellular domain, and are involved in the proteolytic processing of multiple transmembrane proteins, cell adhesion, migration and cell signal transduction (5). ADAM9, a member of the ADAM family, demonstrates proteinase activity which is important in the cleavage of proHB-EGF, known as ectodomain shedding. ADAM9 is also responsible for mediating EGF receptor activity (6) and is capable of regulating E-cadherin and integrins (7,8), demonstrating its important role in cancer cell invasion, migration and metastasis (5–8).

Recently, highly expressed ADAM9 was detected in breast cancer (9), liver cell carcinoma (10), gastric cancer (11), pancreatic ductal adenocarcinoma (12), prostate cancer (13), renal cell carcinoma (14) and cervical squamous carcinoma (15), correlating with cancer progression, metastasis and predicting a shortened survival time in patients (9–15). However, the expression of ADAM9 in human resected lung cancer tissue and its clinical significance remain unclear.

We recently demonstrated that ADAM9 was highly expressed in 39 cases of resected stage I NSCLC tissues compared with normal control lung tissue (16). In the present study, we detect further ADAM9 expression at the protein level in surgically resected stage I NSCLC, attempting to confirm the findings of highly expressed ADAM9 in NSCLC, and to further evaluate its clinical significance, especially with regard to predicting the prognosis of resected stage I NSCLC (abstract was published at the 14th World Conference on Lung Cancer, Amsterdam, 2011) (17).

## Materials and methods

### Patients and tissues

Formalin-fixed, paraffin-embedded tissue blocks were obtained from 64 cases of completely resected NSCLC with mediastinal N2 lymph node dissection who underwent surgery at the Department of Thoracic Surgery, The First Hospital of China Medical University (Shenyang, China) between April 2000 and May 2006. All patients underwent standard lobectomy and mediastinal N2 lymph node dissection. Preoperative examinations of liver ultrasound, brain computed tomography (CT) and bone scintigraphy were performed to exclude the presence of distant metastasis. The following criteria were used to exclude patients: the patients received preoperative chemotherapy or radiotherapy, died within 3 months of surgery or succumbed to a cause other than NSCLC.

The total number of patients in this group (n=64) consisted of 36 males and 28 females who ranged in age between 33 and 76 years old (median age, 60 years). Histological types were determined according to the WHO 2000 classification: 16 cases of squamous cell carcinoma and 48 cases of adenocarcinoma. Postoperative pathological stage was classified according to the UICC and AJCC TNM staging system (3): T1N0M0 stage IA, 24 cases (37.5%); T2N0M0 stage IB, 40 cases (62.5%). Normal control lung tissue was collected from >5 cm away from the lung tumor site in 12 cases of completely resected stage IA lung cancer. The study was approved by the Institutional Review Board (IRB) of the First Hospital of China Medical University.

### Immunohistochemistry staining

Sections (3 *μ*m) were cut from formalin-fixed, paraffin-embedded lung cancer tissue. After baking overnight at 60°C, the sections were dewaxed with xylene and gradually hydrated, and then exposed to 3% H_2_O_2_ for 12 min to quench endogenous tissue peroxidase. Antigen was exposed by heating the sections under high pressure in citrate buffer (pH 6.0), cooked for 2 min and then incubated for 20 min in goat serum. The primary goat polyclonal ADAM9 antibody (AF949) was bought from R&D Systems (Minneapolis, MN, USA) and diluted with PBS (1:50) (12–14,16). The specificity and sensitivity of the antibody used have been well tested (12–14,16). After incubation overnight at 4°C, biotin-labeled rabbit-anti goat IgG (Maixin Bio, Fuzhou, China) was used as a secondary antibody incubated for 20 min at room temperature, followed by Streptavidin-Peroxidase (S-P; Maixin Bio, Fuzhou, China) incubated for 20 min at room temperature. Immunohistochemistry staining was visualized with 3,3′-diaminobenzidine (DAB) system for 2–3 min. Afterwards, the slides were briefly counterstained with hematoxylin. ADAM9-positive NSCLC slides were used as the positive control, and the ADAM9-positive slides in which the primary antibody was omitted during staining were used as the negative control.

### Standard of staining scores

The positive cells were stained from yellow to brown in the cytoplasm and cell membrane. For each section, five fields of vision were selected and total 1000 tumor cells were counted and evaluated (200 tumor cells in each field of vision). The percentage of positive tumor cells was calculated. The staining index was evaluated semiquantitatively as negative, weak, moderate or strong by the multiplication of the staining intensity (A) and the percentage of positive tumor cells (B). The staining intensity was scored as 1, buff; 2, buffy; and 3, puce. The percentage of positive tumor cells was scored as: 0, positive tumor cells <5%; 1, 5–10%; 2, 11–50%; 3, 51–75%; 4, >75%. The total score (immunostaining index) was the multiplication of A and B and was classified as follows: a score of 0 was considered as negative (−), 1–4 was considered weak (+), 5–8 as moderate (++) and 9–12 as strong (+++).

All of the cases were divided by average immunostaining index (mean scores) into two groups, ADAM9 high expression (ADAM9-high) group and ADAM9 low expression (ADAM9-low) group. The immunostaining was evaluated independently by two pathologists who were blinded to the patients’ score.

### Statistical analysis

Statistical analysis was performed using SPSS software version 17.0. A Chi-square test and Fisher’s exact test were used to assess the correlation between clinicopathological characteristics and ADAM9 expression. For the survival analysis, the Kaplan-Meier method was used for a univariate analysis, and the differences in survival curves were assessed with the Log-rank test. The Cox regression model was used for multivariate survival analysis. P<0.05 (two-tailed test) was considered to indicate a statistically significant result.

## Results

### Highly expressed ADAM9 in NSCLC tissue

ADAM9 expression was mainly identified in the cytoplasm. In normal control lung tissue, ADAM9 staining was mainly observed to be negatively or weakly expressed and scores belonged to the ADAM9-low group. The ADAM9-high rate was 0% (0/12) in normal control lung tissues.

**Figure 1:**
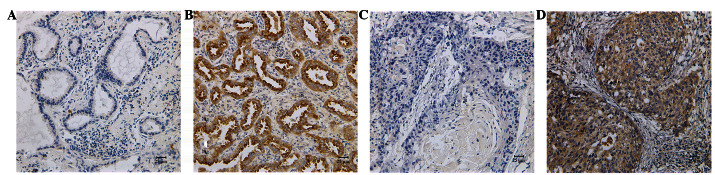
ADAM9 expression in non-small cell lung cancer tissues (S-P method, ×200). (A) ADAM9 low expression in adenocarcinoma of the lung; (B) ADAM9 high expression in adenocarcinoma of the lung; (C) ADAM9 low expression in squamous cell carcinoma of the lung; (D) ADAM9 high expression in squamous cell carcinoma of the lung. ADAM9, a disintegrin and metalloproteinase-9.

In 64 cases of completely resected stage I NSCLC, 53.1% (34/64) demonstrated highly expressed ADAM9 protein, significantly higher when compared with normal control lung tissue (P=0.001). No difference between the ADAM9-high rates between the stage IA and IB groups was identified (P>0.05; Fig. 1).

### Survival analysis: highly expressed ADAM9 predictes shortened survival

**Figure 2:**
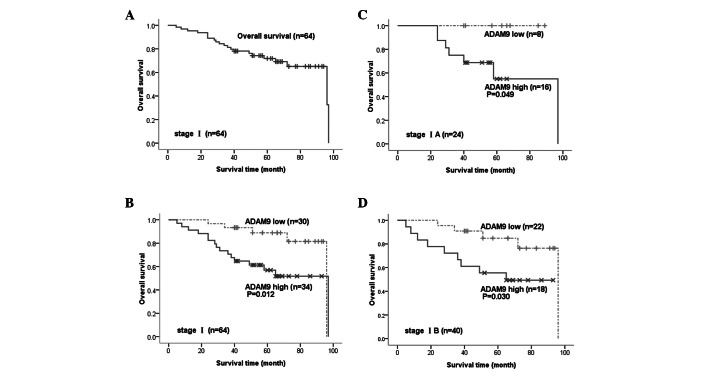
Kaplan-Meier curves of overall survival. (A) Overall survival of all 64 cases of resected stage I non-small cell lung cancer (NSCLC), the 5-year survival rate is 71.8%. (B) Difference between the ADAM9-high and -low groups in 64 cases of stage I NSCLC (P=0.012). (C) Difference between ADAM9-high and -low groups in 24 cases of stage IA NSCLC (P=0.049). (D) Difference between ADAM9-high and -low groups in 40 cases of stage IB NSCLC (P=0.030). ADAM9, a disintegrin and metalloproteinase-9.

The overall 5-year survival rate for the group of 64 completely resected stage I NSCLC cases with performed lobectomy, local hilar (N1) and mediastinal (N2) lymph node dissection was 71.8%. The 5-year survival rate in the ADAM9-low group (30 cases) was as high as 88.9%, however, the 5-year survival rate sharply decreased to 56.9% in the ADAM9-high group (34 cases). The difference between these two groups was statistically significant (P=0.012; Table I, Fig. 2).

In 24 stage IA cases, the 5-year survival rate for the ADAM9-low group (8 cases) was 100%, which sharply decreased to 55.0% for the ADAM9-high group (16 cases). Again, the difference was statistically significant (P=0.049). In the 40 stage IB cases, the 5-year survival rate for the ADAM9-low group (22 cases) was as high as 84.8%, but it decreased sharply to 55.6% for the ADAM9-high group (18 cases), and the difference was statistically significant (P=0.030; Table II, Fig. 2).

The Cox regression model was used for multivariate survival analysis: patient gender, age, smoking status, histological type, pathological stage (IA and IB) and ADAM9 high/low expression were entered into the Cox proportional hazard regression model. The results demonstrated that ADAM9 high/low expression was an independent predictor of prognosis for this group of completely resected stage I NSCLC (HR, 3.385; 95% CI, 1.224–9.360; P=0.019).

## Discussion

Even though ADAM9 has been observed to be highly expressed in numerous solid malignant tumors (9–15), correlating with cancer cells’ invasion, migration, metastasis, involvement of lymph nodes and a worse prognosis (5–15), the expression of ADAM9 at the protein level and its clinical significance in human resected NSCLC cases remains unclear. In NSCLC cell lines, ADAM9 overexpression in A549 cells was shown to increase the ability of adhesion, invasion and metastasis to the brain tissue of nude mice, through modulation of integrin α3β1 function in cancer cells (18). We previously demonstrated that ADAM9 is downregulated when using siRNA silencing hepatoma-derived growth factor (HDGF), inducing inhibition of anchorage-independent growth of NSCLC cells and their capability of migrating through the BD matrigel (19,20), suggesting that ADAM9 participates in the HDGF pathway to promote invasion and metastasis of NSCLC cells. HDGF was shown to be highly expressed in NSCLC and highly expressed HDGF correlated with shortened survival time in NSCLC patients (21). We then preliminarily detected ADAM9 expression at the protein level in the tissue samples of 39 cases of resected stage I–III NSCLC (16), and revealed that ADAM9 was highly expressed in NSCLC tissue, suggesting that ADAM9 may be important in NSCLC growth, invasion and metastasis.

In this study, we confirmed that ADAM9 was significantly more highly expressed in NSCLC when compared with normal control lung tissue (53.1 vs. 0%, P=0.001). Survival analysis revealed that the high expression of ADAM9 predicted a shortened survival in the group of patients with completely resected stage I NSCLC. The 5-year survival rate decreased sharply from as high as 88.9% in the ADAM9-low group to 56.9% in the ADAM9-high group. The difference was statistically significant (P=0.012). Multivariate survival analysis revealed that ADAM9 expression, instead of other factors such as patients’ gender, age, smoking status, histological type and pathological stage (IA and IB), was an independent predictor of prognosis for this group of surgically resected stage I NSCLC cases (HR, 3.385; 95% CI, 1.224–9.360; P=0.019). The results are consistent with the findings that ADAM9 is highly expressed and high levels of ADAM9 expression predict a worse prognosis in other types of solid malignant tumors (9–15), suggesting that ADAM9 is a novel and valuable prognostic biomarker for NSCLC. Adding sub-groups stratified by ADAM9 expression into the lung cancer TNM stage system may also help to supply more accurate information for assessing prognosis.

Furthermore, sub-grouping by ADAM9 expression clearly revealed that the sub-group of ADAM9-high stage I NSCLC, having a 5-year survival rate of 56.9%, which is almost as low as the 5-year survival rate in patients with resected stage II NSCLC (3), should receive further treatment, such as adjuvant chemotherapy, in order to obtain an improved prognosis. ADAM9 may also become a predictive biomarker to help improve the selection of certain stage I NSCLC patients to receive adjuvant chemotherapy or not.

By contrast, for the sub-group of ADAM9-low stage I NSCLC, with low expression of ADAM9, demonstrating a significantly longer survival time, the 5-year survival rate was 88.9% in our study, suggesting that no further adjuvant chemotherapy is necessary for this group of stage I NSCLC, especially considering long-term chemotherapy-associated toxicity (22,23).

Previously, Zhu *et al*(24) reported the use of prognostic signatures to divide NSCLC patients into two groups; a high-risk and a low-risk group. Patients who were predicted a ‘worse’ prognosis benefited significantly from adjuvant chemotherapy; however, the patients who were predicted with ‘better’ prognosis did not benefit from adjuvant chemotherapy, suggesting that sub-grouping by valuable prognostic biomarkers is important in prognosis judgment, especially in aiding the selection of adjuvant chemotherapy (22,23,25). Greater effort should be taken to test more novel and valuable prognostic and predictive biomarkers, such as ADAM9 and HDGF (21), in order to use more simple but accurate methods, to aid decision-making in regard to NSCLC patients receiving personalized adjuvant chemotherapy or not.

To the best of our knowledge, this is the first study describing highly expressed ADAM9 protein in human resected NSCLC tissues predicting a shortened survival. Limited cases have provided us with sufficient evidence to obtain a preliminary conclusion. Using a simple but clinically useful and accurate method, ADAM9 was shown to be a novel prognostic biomarker and may also be a valuable predictive biomarker for adjuvant chemotherapy for completely resected stage I NSCLC patients. In addition, ADAM9 may become a potential target for molecular targeted therapeutics. Further investigations of prospective case-control cohort studies are required to confirm the role of ADAM9 in predicting prognosis and effectiveness of adjuvant chemotherapy for completely resected stage I NSCLC patients.

In conclusion, ADAM9 is highly expressed in NSCLC and highly expressed ADAM9 correlates with shortened survival. ADAM9 expression is an independent prognostic predictor for resected stage I NSCLC, suggesting that ADAM9 is a novel biomarker significantly and independently predicting worse prognosis of resected stage I NSCLC. ADAM9 should also be a valuable predictive biomarker for selection of adjuvant chemotherapy for completely resected stage I NSCLC patients.

## Figures and Tables

**Table I:** Univariate analysis for 64 cases of resected NSCLC.

Variable	n	Proportion (%)	5-year survival (%)	P-value
NSCLC	64	100	71.8	
ADAM9 expression				0.012^a^
Low expression	30	46.9	88.9	
High expression	34	53.1	56.9	
Gender				0.938
Male	36	56.3	75.0	
Female	28	43.7	69.0	
Age (years)				0.736
<60	31	48.4	68.8	
≥60	33	51.6	74.8	
Histological type				0.227
Squamous	16	25.0	87.5	
Adenocarcinoma	48	75.0	67.0	
Pathological stage				0.526
IA	24	37.5	71.3	
IB	40	62.5	71.5	

a P<0.05. NSCLC, non-small cell lung cancer; ADAM9, a disintegrin and metalloproteinase-9.

**Table II:** Highly expressed ADAM9 predicts a worse prognosis for 64 cases of resected NSCLC.

Clinicopathological factors	Cases (n)	5-year survival, %		P-value
		ADAM9 low expression (n)	ADAM9 high expression (n)	
Histological type				
Adenocarcinoma	48	86.5 (16)	57.2 (32)	0.071
Squamous	16	92.9 (14)	50.0 (2)	0.180
Pathological stage				
IA	24	100 (8)	55.0 (16)	0.049^a^
IB	40	84.8 (22)	55.6 (18)	0.030^a^

a P<0.05. NSCLC, non-small cell lung cancer; ADAM9, a disintegrin and metalloproteinase-9.

## Abbreviations:

ADAM9: a disintegrin and metalloproteinase-9;
NSCLC: non-small cell lung cancer;
EGF: epithelial growth factor;
CT: computed tomography;
AJCC: American Joint Committee on Cancer;
UICC: Union Internationale Contre le Cancer;
S-P: streptavidin-peroxidase;
DAB: 3,3′-diaminobenzidine;
HDGF: hepatoma-derived growth factor
